# Differential diagnosis and treatment of acute cauda equina syndrome in the human immunodeficiency virus positive patient: a case report and review of the literature

**DOI:** 10.1186/s13256-016-0902-y

**Published:** 2016-06-06

**Authors:** George Panos, Dionysios C. Watson, Ioannis Karydis, Dimitrios Velissaris, Marina Andreou, Vasilis Karamouzos, Maria Sargianou, Antonios Masdrakis, Paraskevi Chra, Lavrentios Roussos

**Affiliations:** 1 Special Infections Unit, 2nd Internal Medicine Clinic, 1st Ι.Κ.Α. Penteli General Hospital, Melissia, Athens, Greece; 2 Department of Infectious Diseases, Patras University General Hospital, 26504 Rion, Patras Greece; 3 Internal Medicine Department, University Hospital of Patras, 26504 Rion, Patras Greece; 4 Department of Microbiology, Benakio-Korgialenio Hospital, 1 Erythrou Staurou Street, 11526 Athens, Greece; 5 Neurosurgery Clinic, Κ.Α.Τ. Hospital, 2 Nikis Street, 14561 Kifissia, Athens Greece

**Keywords:** HIV, Cauda equina syndrome, Lumbar disk fragment, CMV, Myeloradiculopathy, Mycobacterium tuberculosis

## Abstract

**Background:**

Acute cauda equina syndrome is an uncommon but significant neurologic presentation due to a variety of underlying diseases. Anatomical compression of nerve roots, usually by a lumbar disk hernia is a common cause in the general population, while inflammatory, neoplastic, and ischemic causes have also been recognized. Among human immunodeficiency virus (HIV) infected patients with acquired immunodeficiency syndrome, infectious causes are encountered more frequently, the most prevalent of which are: cytomegalovirus, herpes simplex virus 1/2, varicella zoster virus, and *Mycobacterium tuberculosis* infections. Studies of cauda equina syndrome in well-controlled HIV infection are lacking. We describe such a case of cauda equina syndrome in a well-controlled HIV-infected patient, along with a brief review of the literature regarding the syndrome’s diagnosis and treatment in individuals with HIV infection.

**Case presentation:**

A 36-year-old Greek male, HIV-positive patient presented with perineal and left hemiscrotal numbness, lumbar pain, left-sided sciatica, and urinary incontinence. Magnetic resonance imaging of the patient’s lumbar spine revealed intrathecal migration of a fragment from an intervertebral lumbar disk exerting pressure on the cauda equina. A cerebrospinal fluid examination, brain computed tomography scan, spine magnetic resonance imaging, and serological test results were negative for central nervous system infections. Our patient underwent emergency neurosurgical spinal decompression, which resolved most symptoms, except for mild urinary incontinence.

**Conclusions:**

Noninfectious etiologies may also cause cauda equina syndrome in HIV-infected individuals, especially in well-controlled disease under antiretroviral therapy. Prompt recognition and treatment of the underlying cause is important to minimize residual symptoms. Targeted antimicrobial chemotherapy is used to treat infectious causes, while prompt surgical decompression is favored for anatomical causes of cauda equina syndrome in the HIV-infected patient.

## Background

Cauda equina syndrome (CES) is a polyradiculopathy resulting from nerve compression or inflammation of the lower spinal cord, which commonly presents with motor and sensory abnormalities of the lower limbs, along with bladder and sphincter dysfunction. Numerous causes of CES have been reported to date, including spinal disk herniation, trauma, spinal stenosis, iatrogenic causes, neoplasms, ischemia, inflammation, and infections [1]; an etiology for a portion of cases cannot be identified. In patients with advanced human immunodeficiency virus/acquired immunodeficiency syndrome (HIV/AIDS), central nervous system (CNS) infections, usually due to cytomegalovirus (CMV) or *Mycobacterium tuberculosis* (MTB), comprise the most common causes of CES [2–5]. However, noninfectious processes may also lead to CES in individuals infected with HIV, especially when the disease is well controlled, as in the case report that follows. Prompt diagnosis of the syndrome, identification of the underlying cause, and appropriate treatment are crucial, since progression is rapid and prognosis can sometimes be unfavorable if specific treatment is delayed, especially in the context of HIV infection.

## Case presentation

A 36-year-old Greek male, HIV-positive patient was admitted to the Neurology Department due to perineal and left hemiscrotal numbness, lumbar pain, left-sided sciatica, and urinary incontinence; his symptoms began 5 days prior to admission, and were not accompanied by fever. The patient’s occupational history was significant for frequent weight-lifting during his daily duties as an orderly. He had been on a triple antiretroviral regimen with didanosine (ddI), d4T (stavudine), and efavirenz for the past 6 years, while diagnosis of HIV was made 12 years earlier. Recent testing reported a CD4^+^ cell count of 1036 cells/mL and an undetectable HIV viral load (<50 copies/mL); the Center for Diseases Control (CDC) stage of his disease was C3.

The neurological examination revealed decreased strength of the left gastrocnemius and absence of the left ankle jerk reflex, while his plantar reflexes were indifferent bilaterally. The remaining physical examination was noncontributory.

Given that the patient’s neurological signs and symptoms were indicative of CES, a magnetic resonance imaging (MRI) scan of his lumbar spine was initially performed. Imaging revealed left dorsolateral intervertebral disc herniation of L3-L4, prominent dorsomedian intervertebral disc herniation of L5-S1, and a small fragment within the vertebral canal (at S1) that was exerting pressure on cauda equina fibers. The latter finding was compatible with a fragment originating from the L5-S1 intervertebral disk. A brain computed tomography (CT) scan, cerebrospinal fluid (CSF) examination, and serological test results for CNS infections, including CMV and herpes simplex virus 1/2 (HSV-1/2) were negative. Further testing for infectious causes would have been pursued in the absence of an obvious anatomical cause of the observed syndrome.

Because of the acute onset of CES in our patient, emergency neurosurgical spinal decompression was deemed necessary. The procedure was successful overall, leading to resolution of most neurological symptoms, with the exception of a persistent, mild urinary incontinence. Specifically, early postoperative neurological findings were largely limited to the left lower limb and perineum, and included: slightly decreased lower limb muscle strength (L5-S1 myotomes), mild sensory impairment on the lateral surface of his foot (L5 dermatome), and mild paresthesia of his buttocks, left hemiscrotum, and dorsolateral thigh. Two-point discrimination was 1.5–2 cm, with a mild decrease on his left side. Vibration sensation in the affected regions was normal, and his plantar reflexes were flexor bilaterally.

Neurological follow-up performed 1 week later revealed physiological muscle strength, no sensory deficits, and partial recovery of his bladder dysfunction. Urological symptoms have been previously described in the literature as presenting a delayed recovery following decompression surgery [6]. Although intervertebral lumbar disc herniation with posterior migration of a sequestered disk fragment has been previously reported as a rare cause of CES in immunocompetent patients [7], this is the first case report in an HIV-positive patient.

## Discussion

### Differential diagnosis

Despite the relative rarity of CES among HIV-positive patients, prompt diagnosis and treatment of the syndrome is crucial due to the potential of persistent debilitating symptoms [8] (such as permanent loss of bowel/bladder control and paralysis of the legs). It is, thus, necessary to perform an immediate and thorough clinical, imaging, and laboratory investigation in every HIV-positive patient whose symptoms are suggestive of the syndrome in order to identify its cause. An MRI scan of the lumbar spine should be performed within the first few hours of symptom onset [9]. This aims to quickly identify many of the causes of the syndrome (Table 1), such as simple protrusion of the intervertebral disc, compression of the cauda equina, or focal inflammation (for example, caused by infection) [8].

**Table 1:** Causes of cauda equina syndrome in human immunodeficiency virus positive patients

Anatomical compression	Spinal infection (especially in advanced HIV/AIDS)	Other causes
Abscess [32, 33]	Cryptococcus [34]	Connective tissue disease [24, 35]
Spinal hematoma (for example, from trauma) [36]	Cytomegalovirus [37]	Iatrogenic adhesive arachnoiditis [38]
Intervertebral disk hernia [28]	Epstein-Barr virus [39]	HIV (idiopathic cauda equina syndrome) [14]
Intrathecal disk fragment [7]	Herpes simplex virus (mainly HSV-2) [19]	Spinal ischemia [40]
Pathologic spinal fracture (for example, due to osteoporosis or neoplasia) [41]	*Treponema pallidum* [22]	
Spinal neoplasia (especially lymphoma) [42–44]	Schistosoma [45]	
Vertebral canal stenosis [46]	Toxoplasma [47]	
	*Mycobacterium tuberculosis* [20]	
	*Varicella zoster* virus [48, 49]	

*HIV* human immunodeficiency virus, *AIDS* acquired immunodeficiency syndrome

In the general population, CES usually results from compression of spinal nerve roots due to intervertebral disk hernias, vertebral canal stenosis, or spinal neoplasms [1]. Intervertebral lumbar disc herniation with posterior migration of a disc fragment has been reported as a rare cause of the syndrome in otherwise healthy persons [7, 10, 11]. As for noncompressive causes, ischemia and spinal arachnoiditis have been described [1]. These processes could be the cause of CES in HIV-infected individuals, especially in the setting of well-controlled infection under antiretroviral therapy.

The differential diagnosis of CES in immunocompromised patients with HIV infection includes several more, mostly infectious, causes (Table 1). In HIV-infected individuals with advanced HIV/AIDS, the syndrome may present as an acute polyradiculopathy [12, 13]. When this occurs, it is often caused by CNS infection that affects the cauda equina or conus medullaris. CMV and MTB are the most common infectious causes among HIV-positive patients [5, 13, 14], which can be in the context of concurrent infection of the retina and other organs [2, 13].

In order to rule out CMV CNS infection, a lumbar puncture may be performed for CSF analysis, along with serological and molecular testing. CSF laboratory findings suggestive of CMV CNS infection include high cell count with unaltered polymorphonuclear cells (usually >60 %), decreased glucose (<50 % of serum measurement), and significantly increased protein levels, whereas neoplastic cells are absent [2, 13]. Nevertheless, reports of normal CSF findings in CMV infections have also been published [13]. Thus, polymerase chain reaction (PCR) of the CSF for CMV DNA is also required before CMV infection is excluded in HIV-positive patients (92 % sensitivity and 94 % specificity) [15], whereas branched chain DNA (bDNA) assay for CMV DNA and immunoperoxidase staining for CMV antigen can also be considered [16, 17]; CSF viral cultures are less useful for initial diagnosis, as they lack sensitivity [13, 15]. In addition to molecular testing, MRI with intravenous gadolinium contrast may be necessary to detect neural CMV lesions [18].

Myeloradiculitis is usually a complication of tuberculous meningitis or HSV-2 CNS infection that can also lead to CES (even in successfully treated patients) [4, 19] and yields negative CSF examination results [20, 21]. Depending on patient history and locally endemic diseases, ruling out HSV-1/2, *varicella zoster* virus (VZV), and *Mycobacterium tuberculosis *(MTB) should also be considered; molecular testing may also be necessary in these cases, as other testing can be nondiagnostic [4, 19]; other, more rare causes should be considered on an individualized basis.

Also, impaired immune response in HIV-positive patients with syphilis can cause an atypical, aggressive form of neurosyphilis, which can manifest as painful polyradiculopathy [22]; rapidly progressive, asymmetrical sensorimotor abnormalities of the lower limbs, bladder and sphincter are the hallmark of this form of neurosyphilis. In the full-blown syndrome, the patient may present with sensory loss of the lower body’s dorsal surface, bilateral sciatic nerve pain, paresthesia, decreased strength of the lower limbs or chronic flaccid paraplegia, absence of deep tendon reflexes, and bladder/sphincter dysfunction [1]. Lymphomas have also been reported to cause CES in HIV-positive patients [5, 23]. Finally, primary CES in these patients is a benign, idiopathic form of the syndrome that has a slow clinical progression, mild neurologic deficits, and a generally better prognosis, which may explain those cases where no anatomical causes, infectious agents or neoplastic cells can be identified [24]. CSF examination results in the primary syndrome reveal moderate mononuclear pleocytosis and mildly increased protein levels.

A systematic meta-analysis of more than 200 individual cases of CES found that the majority (84 %) of patients experience a progressive development of symptoms, beginning with sensory-motor findings in the lower extremities (early CES) [25]. Bowel and sphincter symptoms tend to develop later on, in what the authors of the study describe as “incomplete CES” and “CES in retention” [25]. Thus, suspecting CES when bilateral lower limb and/or perineal sensorimotor findings are present could lead to more rapid diagnosis and subsequent management in both well-controlled and advanced HIV infection.

### Treatment

Treatment should always be specific, unless the cause of the syndrome cannot be identified. In the case of the latter, short-term alleviation of symptoms and long-term complete neurological recovery of the patient are sought.

In the case of lumbar disk herniation causing CES in HIV-positive patients, surgery is considered more effective than medical management in the short term [26]. In the single relevant study, no significant difference in the response of HIV-infected individuals to surgical treatment of lumbar disk herniation was found, as compared to uninfected patients [27]. It is estimated that 1–3 % of all patients with intervertebral lumbar disc herniation may manifest with CES [28, 29]. These cases of the syndrome, as well as those caused by other anatomical compressive phenomena, undergo surgical decompression that is performed within the first 24 hours of symptom presentation, in order to achieve maximum postoperative neurological recovery [29, 30]. Delayed surgical decompression (more than 48 hours after symptom onset versus treatment within 24–48 hours) has been correlated with a much higher rate of postoperative bladder and anal sphincter dysfunction, serious motor deficits, sexual dysfunction, and persistent pain [29].

In HIV-positive patients, when CES is due to CMV CNS infection, treatment may consist of intravenous administration of ganciclovir for 2–3 weeks; foscarnet can also be added to the treatment regimen when there is an increased risk for treatment failure, as in patients who have previously received ganciclovir, or in those with persistent polymorphonuclear pleocytosis, decreased glucose, and/or detection of CMV in serial CSF tests [3, 31]. In ganciclovir-resistant cases, foscarnet infusion can be performed alone or in combination with cidofovir. Progression of neurological symptoms is usually controlled within 2 weeks of treatment, while serious defects may persist even after treatment completion [2, 12]. Contrarily, in idiopathic CES spontaneous resolution of the syndrome is most often the case [12].

Initiation of empirical treatment may be considered in advanced HIV/AIDS patients with CES until the etiology is revealed, in order to prevent irreversible progression of neurological deficits [4]. Given the high occurrence of *Herpesviridae* and MTB infections leading to CES among HIV-infected patients, it may be reasonable to consider ganciclovir, acyclovir, and antimycobacterials in empirical regimens, while patient history and findings will further guide the empirical treatment selection [4].

## Conclusions

It is important for acutely presenting CES to be suspected in patients with bilateral lower limb and/or perineal sensorimotor deficits, so as to mitigate permanent neurological damage. In working up HIV-positive patients, it can be important to exclude infectious causes of the syndrome, but differential diagnosis should also include etiologies leading to CES in the general population, especially in patients with well-controlled HIV infection.
